# Current understanding of the relationship between cervical manipulation and stroke: what does it mean for the chiropractic profession?

**DOI:** 10.1186/1746-1340-18-22

**Published:** 2010-08-03

**Authors:** Donald R Murphy

**Affiliations:** 1 Rhode Island Spine Center, 600 Pawtucket Avenue, Pawtucket, RI 02860, USA; 2 Department of Community Health, Alpert Medical School of Brown University, Providence, RI, USA; 3 Department of Research, New York Chiropractic College, Seneca Falls, NY, USA

## Abstract

The understanding of the relationship between cervical manipulative therapy (CMT) and vertebral artery dissection and stroke (VADS) has evolved considerably over the years. In the beginning the relationship was seen as simple cause-effect, in which CMT was seen to cause VADS in certain susceptible individuals. This was perceived as extremely rare by chiropractic physicians, but as far more common by neurologists and others. Recent evidence has clarified the relationship considerably, and suggests that the relationship is not causal, but that patients with VADS often have initial symptoms which cause them to seek care from a chiropractic physician and have a stroke some time after, independent of the chiropractic visit.

This new understanding has shifted the focus for the chiropractic physician from one of attempting to "screen" for "risk of complication to manipulation" to one of recognizing the patient who may be having VADS so that early diagnosis and intervention can be pursued. In addition, this new understanding presents the chiropractic profession with an opportunity to change the conversation about CMT and VADS by taking a proactive, public health approach to this uncommon but potentially devastating disorder.

## Introduction

Cervical manipulative therapy (CMT) and vertebral artery dissection and stroke (VADS) have been linked in controversy for at least 75 years [1]. At the center of the controversy have been neurologists and other medical practitioners who have often perceived VADS to be a relatively frequent complication to CMT [2,3] and chiropractors, who have generally perceived VADS after manipulation to be exceedingly rare [4-6]. Others have been involved as well [7,8]. Starting with isolated case reports and culminating in four case-control studies, our understanding of the relationship between CMT and VADS has evolved considerably. The purpose of this commentary is to present an overview of the history of this relationship and to discuss how the chiropractic profession and other professionals who use manual therapy can move forward and focus on the wellbeing of patients and the public while avoiding defensiveness.

There are several pathophysiologic processes that can lead to stroke, such as atherosclerosis, hemorrhage secondary to aneurism or arteriovenous malformation, and arterial dissection. Arterial dissection is a specific process in which a tear occurs in the wall of the involved artery [9]. Cervical artery dissection is a general term for dissection that involves either the carotid artery (carotid artery dissection) or vertebral artery (vertebral artery dissection). As vertebral artery dissection has been found to have an association with visits to chiropractic physicians, this commentary primarily focuses on vertebral artery dissection. However, the terms cervical artery dissection and carotid artery dissection are used in certain instances in which both carotid artery dissection and vertebral artery dissection or carotid artery dissection alone is being referred to.

### The evolution of our understanding of the relationship between cervical manipulation and vertebral artery dissection - case reports, surveys, biomechanical studies, case reviews

The awareness of a temporal relationship between cervical CMT and VADS began with a series of case reports published over a period of several years [10-22]. In a number of these studies, the treating practitioner was incorrectly identified as a chiropractor [23]. These studies reported on cases of patients who developed VADS some time after receiving CMT. Generally in these reports the CMT was described as the cause of the dissection. In addition to CMT a number of reports attributed the cause of VADS episodes to a number of other mechanical events which preceded the VADS episode [24].

Later came a series of retrospective surveys. The first of these was a survey of the 367 members of the Swiss Society for Manual Medicine who were asked to recall over the course of their career (minimum 2 years, maximum 33 years, mean 8.1 years) how many CMTs they had provided and how many complications had occurred following CMT [25]. They estimated the rate of "slight neurological complications" to be 1:40,000 and the rate of "important complication" to be 1:400,000. Next was a survey of California neurologists who were members of the American Academy of Neurology [2]. In this study, recipients of the survey were asked to recall over the previous two years how many "neurologic complications following chiropractic adjustment", including radiculopathy, myelopathy and VADS, they had encountered. The authors reported a 37% response rate. Twenty-one percent reported at least one case of stroke. This was followed by a 10-year retrospective survey of chiropractors [26] in which the then-226 members of the Danish Chiropractors Association were surveyed (response rate 54%) in an effort to determine the incidence of "cerebrovascular incidents" between 1978-1988. From these data they estimated an incidence of one case per 362 chiropractor years and one case per 1.3 million cervical treatment sessions.

Later, a biomechanical study was performed by Symons, et al [27]. They used five unembalmed cadavers and exposed their cervical spines to movements similar to those that occur during clinical examination of range of motion as well as high-velocity, low amplitude CMT using a combined lateral flexion-rotation maneuver. This CMT was applied separately to the upper, middle and lower cervical spine. They measured the strain on the vertebral artery during these maneuvers. The arteries were then harvested and stretched to mechanical failure. They found that during ROM testing the strain to the vertebral artery was 1.2% to 12.5% greater than that at rest (the amount of strain varied according to the direction of movement applied). During CMT the average strain was 6.2% greater than that at rest. Finally, they found that mechanical failure did not occur until average strains of 139%-162% greater than that at rest. The authors concluded that the strain applied to the vertebral artery during CMT was unlikely to tear or otherwise mechanically disrupt a normal vertebral artery [27]. Limitations of this study were pointed out in two subsequent letters to the editor [28,29], including that fact that this study assessed gross failure of the artery but not other possible mechanisms by which CMT may cause vertebral artery dissection.

Other notable studies were published as well. Haldeman, et al [30] retrospectively reviewed 23 cases of VADS that occurred following CMT, utilizing data from a Canadian chiropractic malpractice insurance carrier over a 10-year period. From these cases they estimated the number of neurologists and chiropractors who were directly involved in each case. They calculated that one in 48 chiropractors was exposed to such cases, in comparison to one in two neurologists. They concluded that this selection or referral bias likely explained why neurologists tend to perceive VADS after CMT to be far more common than do chiropractors. Haldeman, et al [31] performed a retrospective review of 64 cases of VADS temporally related to CMT. They found no factors in the history or examination that would assist the physician in identifying the individual at risk of VADS after CMT. These authors concluded "Cerebrovascular accidents after manipulation appear to be unpredictable and should be considered an inherent, idiosyncratic, and rare complication of this treatment approach" [31].

However none of the study designs discussed above are adequate to assess risk and to investigate a causal relationship between CMT and VADS. Descriptive studies such as case reports and case series are limited due to the absence of a comparison group [32,33]. For example, in a case study in which a patient's headaches are reported to have improved after CMT, there is no way to determine whether the headaches would have improved without the CMT. Likewise, if an individual experiences an adverse event (e.g. VADS) following a treatment (e.g. CMT) or any other exposure there is no way to determine from a case report or case series whether that adverse event would have happened regardless of the treatment or exposure. To undertake an assessment of risk one must use one of three study designs:

Randomized, controlled trial (RCT): this is a design in which individuals are randomly assigned to one of two or more groups. Each group is provided a treatment, placebo, sham or no treatment and the outcomes of the groups are compared. The RCT is considered the Gold Standard for assessing treatment efficacy but is rarely used for risk assessment [34].

Prospective cohort study: this is a study which follows two or more groups over time, one of which is exposed to a certain treatment or condition of interest and the other of which is not, and compares them for a particular outcome [34,35]. This design works well if the condition of interest is relatively common, such as heart disease. Perhaps the most well-known cohort study is the Framingham Heart Study (http://www.framinghamheartstudy.org/participants/original.html accessed 3 June 2010), which has tracked the rate of heart disease and its association with various risk profiles in an original cohort of 5,209 people since 1948 prospectively over time. The prospective cohort design does not work well for studying a rare disease such as VADS, because one could follow thousands of patients for many years and potentially never come across a case of VADS.

Case-control study: this is the best research design for assessing the risks associated with a rare disorder such as VADS [33-35]. The case-control design compares a group of people who already have the outcome of interest to a similar group of people who do not. The researchers compare the two groups for exposures to a certain treatment or other factor prior to development of disease.

Using the case-control study design allows researchers to gain insight into whether the apparent relationship between an exposure (e.g., CMT) and an outcome (e.g., VADS) that is observed in case reports or case series is a true association, and allows causal inferences to be made [34]. It does this in the case of the relationship between CMT and VADS by identifying individuals who already have VADS and comparing them to a matched control group of individuals without VADS with regard to exposures to CMT prior to developing VADS. Essential to minimizing bias in case-control studies is appropriate matching of cases and controls [35]. That is, the control group should be comparable to the "case" group. Reduction of bias in this regard is sometimes addressed by using a case-crossover design [36] in which cases serve as their own controls. This helps to better match the groups which reduces bias by better controlling for confounding variables [36].

### Case-Control Studies on the Relationship between CMT and VADS

Four case-control studies have investigated the relationship between CMT and VADS. The first was by Rothwell, et al. [37] This was a six year study performed in Ontario that compared 582 individuals who experienced VADS (cases) with 2328 individuals with no history of stroke (controls). They found that cases aged under 45 were five times more likely to have had a visit to a chiropractor within one week of their stroke. In individuals 45 or older, there was no difference between cases and controls. The second case-control study was that of Smith, et al. [38] This was a six year study performed in two academic stroke centers. They compared 51 patients with stroke related to cervical artery dissection (25 involving the vertebral artery, 26 involving the carotid artery) with 100 patients with other types of stroke. Multivariate analysis found that patients with VADS were six times more likely than controls to have seen a chiropractor within 30 days of stroke. They concluded that CMT is an independent risk factor for VADS.

This was followed by a study by Dittrich, et al [39] in which 47 cases of either VADS or stroke related to carotid artery dissection were compared to 47 controls with other types of stroke. They looked at a variety of "mild" mechanical events that were potential risk factors (heavy lifting, mild direct neck trauma, mild indirect neck trauma, sexual intercourse, jerky or abnormal head movement, athletic activity, CMT) and their relationship to cervical artery dissection individually and as a group. They used seven days as the time period between the mechanical event and stroke for all the potential risk factors except CMT, for which they used a 30 day cutoff. They did not explain the reason for their use of different cutoffs. They found no statistically significant association between any individual mechanical event, including CMT, and cervical artery dissection. They did report a significant association between the mechanical factors as a group and cervical artery dissection. They also stated "our results indicate only a weak association of CMT with CAD, which might, however, be important in the pathogenesis". Unlike the previous studies, they included patients with VADS and stroke related to carotid artery dissection as a group and did not specifically assess the relationship between CMT and VADS.

Two of these three case control studies indicated a clear association between visits to a chiropractor and VADS (though not between visits to a chiropractor and carotid artery dissection). Two possible explanations emerged from these data. One was that CMT can cause VADS in certain susceptible individuals, and that there was no way to predict, or screen for, the individual who was at risk of "post-manipulative stroke" [40-42]. Another, which was pointed out in both the Rothwell, et al paper [37] and the Smith, et al paper [38], was that patients with early symptoms of VADS (neck pain and/or headache) sought care from a chiropractor and, subsequent to the chiropractic visit, went on to experience a stroke independent of the application of CMT [24]. These case-control studies were not able to substantiate either of these theories.

Following these studies was a paper that attempted to answer the question, "Does cervical manipulative therapy cause vertebral artery dissection and stroke?" [43] by reviewing the literature up to that point and "[using] Sir Bradford Hill's criteria for causation as well as the strength of the research designs to present and evaluate the evidence for or against a causal relationship". This study concluded that the criteria of temporality (purported cause preceded effect), dose-response (higher rates of exposure associated with higher rates of disease), consistency of association and biological plausibility supported a cause-effect relationship between CMT and VADS. The criteria of strength of association (the size of the relative risk), specificity (one cause leading to one effect) and analogy (an analogous cause-effect relationship already established for a similar exposure and disease) were deemed equivocal or not in support of a cause-effect relationship between CMT and VADS. It was deemed that the criterion of reversibility (reduction in exposure leading to a reduction in rate of disease) could not be adequately satisfied in answering the posed question. They concluded that their analysis "support(s) weak to moderate strength of evidence for causation between CMT and VAD and associated ischemic stroke, especially in young adults" and called for research "which would employ superior study designs" [43]. They did not discuss the potential confounding factor discussed in the Rothwell, et al [37] and Smith, et al [38] papers of neck and head pain patients presenting to chiropractors with a dissection already in progress.

Triano and Kawchuk [44], in a monograph published by a chiropractic malpractice insurer, applied the same Bradford Hill criteria in an attempt to answer the same question as Miley, et al [43] and came to the conclusion that these criteria do not support a causal relationship between CMT and VADS.

Finally, there is the most recent and largest case-control study by Cassidy, et al. [45] These authors attempted to respond to the need for more rigorous research in this area by adding two elements that were not utilized in previous case-control studies. First, they used a standard case-control design but added a case-crossover design in which cases served as their own controls. Second, they attempted to answer the question raised by the previous case-control studies regarding whether the association found in the Rothwell, et al [37] study was related to CMT being an independent causative factor in VADS or whether patients with VADS after manipulation had a dissection in progress which led to the chiropractic visit. They did this by including not only visits to chiropractors within 30 days but also visits to primary care physicians within the same time period.

This study involved 109,020,875 person-years of observation over a period of nine years. The cases were 818 patients with VADS and the controls were 3164 individuals without stroke. The case-crossover involved four random control periods amongst the individuals in the VADS group prior to their stroke. As with the Rothwell, et al study, [37] they found an increased association between visits to a chiropractor within 30 days and VADS (OR 1.37; 95% CI 1.04-1.91 from the case crossover analysis) in individuals under 45 years of age, but no association in individuals 45 years of age or older. However, they also found an association between visits to primary care physicians and VADS. This association was found both in patients under 45 (OR 1.34; 95% CI 0.94-1.87 from the case crossover analysis) and in those 45 and older (OR 1.52; 95% CI 1.36-1.67 from the case crossover analysis). Another difference between this study and previous case-control studies is that Cassidy, et al compared the association of visits to chiropractors and primary care physicians for complaints related to neck pain or headache with those without neck pain or headache. They found substantially greater associations between visits to both practitioners and VADS when the visits involved neck pain or headache.

It is commonly assumed that if VADS occurs immediately or soon after CMT a clear causal relation is established [46,47]. Cassidy, et al [45] examined this assumption as well and found that the odds of stroke occurring within 24 hours of a visit to a primary care physician was virtually the same as stroke occurring within 24 hours of a visit to a chiropractor [45].

Cassidy, et al [45] point out the limitation of their use of administrative data and investigated this by performing a sensitivity analysis using various positive predictive values for stroke diagnosis. They found that this did not change the study's conclusions.

Therefore, based upon the best current evidence, it appears that there is no strong foundation for a causal relationship between CMT and VADS. The most plausible explanation for the association between CMT and VADS is that individuals who are experiencing a vertebral artery dissection seek care from a chiropractic physician or other manual practitioner for relief of the neck pain and headache *that results from *the dissection. Sometime after the visit the dissection proceeds along its natural course to produce arterial blockage, leading to stroke. This natural progression from dissection to stroke appears to occur independent of the application of CMT.

### Do chiropractors and other practitioners of manual therapy not have to worry about VADS?

The weight of the evidence currently suggests that the most likely explanation for the occurrence of VADS following CMT is that a patient with neck pain and/or headache arising from the arterial dissection seeks the care of a chiropractic physician or other practitioner of manual therapy for relief from this pain, and sometime after this visit the condition independently progresses to a full stroke. It appears that this progression to stroke occurs as a result of the natural history of VADS, although one has to be open to the possibility that this inevitable progression may be hastened by the CMT, so that it occurs sooner than it would have without CMT. In addition, there have been cases reported in which an individual without neck pain or headache has developed VADS after receiving CMT [31]. It is not known how often this occurs after seeing a PCP. Therefore, one would have to be open to the possibility that CMT may precipitate a vertebral artery dissection in a susceptible individual who is not currently having a dissection. However, if this is a possibility, it would have to be considered so rare that a case-control and case crossover study covering over 109,000,000 person-years failed to detect it [45]. Also, in 20% of cases of VADS the individual does not have neck pain or headache and in a very small percentage of patients vertebral artery dissection can occur in a person who has no symptoms of any kind [48]. Thus, in cases in which an asymptomatic individual experiences VADS after CMT it is not clear whether manipulation was a cause or contributing factor to the dissection or whether the patient had an asymptomatic arterial dissection prior to the chiropractic visit.

Thus, the concern for the chiropractic physician and other manual practitioner has shifted. Previously the focus had been on trying to "screen" for a patient who is "at risk" of a rare "complication to CMT" [49-52]. However, multiple publications have pointed to the lack of reliability of screening in the clinic for risk of an episode of VADS that has not yet occurred [42,50,53,54]. Also, current evidence indicates that VADS is not a "complication to CMT" *per se*. That is, the weight of the evidence suggests that CMT is not a cause of VADS (although, as stated earlier, it is possible that in incalculably rare cases CMT may precipitate dissection in a person who already has susceptibility to dissection) but is incidental to it, with the link between the two being the presence of neck pain and/or headache. The issue for practitioners now is one of differential diagnosis. The responsibility of the practitioner is not to attempt to identify the patient who is at risk of "post-manipulative stroke", but to attempt to identify the patient who is having a dissection in progress so appropriate referral can be made.

Certainly, in many cases there are no clear signs or symptoms that can serve to alert the practitioner to the possibility of VADS. In addition, some of the early symptoms of VADS such as dizziness, vertigo, imbalance, nausea and tinnitus are common in patients without VADS who present to practitioners who use manipulation and other forms of manual therapy (as well as PCPs). However there likely are those cases in which history and examination may be useful in identifying the patient with true VADS.

The most common initial symptoms of VADS are neck pain and/or headache [9,55]. Neurologic symptoms and/or signs can begin to manifest shortly after the onset of pain, particularly after the development of headache [56-58]. In addition, the progression from neck pain and headache to full stroke is not always sudden - there is often a period in which subtle signs and symptoms may develop prior to the development of fully manifested stroke [59]. In addition, it is important for the practitioner, in cases in which there are no detectable signs or symptoms of VADS but in which the patient develops these in the office after manipulation, to take appropriate steps to respond to this medical emergency.

### Historical Factors Suggestive of the Possibility of VADS

It has been suggested that individuals with VADS have a genetic predisposition however this is not clear and a recent systematic review of the literature indicated that more research is needed to clarify whether genetics plays a role in the pathogenesis of VADS [60]. Individuals with known connective tissue diseases, such as autosomal dominant polycystic kidney disease, Ehlers-Danlos Type IV, Marfan Syndrome or fibromuscular dystrophy, are at increased risk of developing VADS [44]. Therefore the presence of one of these disorders should raise the clinician's level of suspicion. However the majority of patients with VADS do not have any of these diseases [48,60], so their absence does not rule out the possibility of VADS.

In addition, an association has been found between history of migraine headache and VADS [61]. However, as migraine is a fairly common disorder and VADS is rare, the vast majority of migraineurs will never have VADS, thus limiting migraine history as a means to "rule in" the likelihood of VADS. Plasma homocysteine concentration has been found to have a weak association with VADS [61], however the sensitivity and specificity of this finding is unknown thus routine testing for this in patients with neck pain and/or headache cannot be justified. Weak association has also been found between VADS and recent infection [61]. As was the case with migraine headache, however, the vast majority of individuals who develop some type of infection will not have VADS.

In approximately 80% of patients the initial symptom of VADS is neck pain with or without headache [48]. The headache is typically occipital, occipito-temporal or frontal in location and usually unilateral. It is common for the pain to be described as the worst the patient has ever experienced, but it is important to note that only approximately half of patients who are previous headache sufferers describe the pain as different from their usual headaches [55]. The classic recommendation regarding the detection of signs and symptoms suggestive of VADS is the "5 Ds And 3 Ns". That is, diplopia, dizziness, drop attacks, dysarthria, dysphagia, ataxia, nausea, numbness and nystagmus. This is a good general rule, however it must be remembered that many patients will not have these signs and symptoms early in the process and when they do manifest they may be subtle and may not be volunteered by the patient. So careful questioning may be necessary to detect their presence. (see Table 1)

**Table 1:** Questions the practitioner may ask in seeking the "5 Ds And 3 Ns" principle (though nystagmus is investigated on examination)

	blurred vision?
	
	double vision?
	
	trouble swallowing or speaking?
	
Have you been experiencing:	dizziness?
	
	fainting spells?
	
	nausea?
	
	trouble with walking or balance?
	
	numbness in your hands or feet?

In a patient with sudden onset of severe unilateral neck pain and headache, particularly in the presence of neurologic symptoms, careful examination is advised and watchful waiting or further investigation should be considered.

### Examination

In any patient who presents with new onset of neck pain and/or headache, neurologic examination is warranted. The entire central and peripheral nervous system can be screened on examination in two minutes or less. The "2-minute neurologic exam" is presented in Table 2. Positive findings in response to any of the examination procedures warrant further investigation. In the context of VADS, examination signs related to brainstem or cerebellar involvement, such as cranial nerve dysfunction, nystagmus, difficulty with tandem walking, dysmetria, intention tremor or dysdiadochokinesia should be particularly watched for.

**Table 2:** The 2-Minute Neurologic Examination

Heel, toe and tandem walking	Sensory of the extremities
	
Romberg's position	Motor of the extremities
	
Visual fields	Reflexes of the extremities
	
Pursuit external ocular movement	Plantar response
	
Sensory of the face	Rapid alternating movements
	
Motor of the face	Heel to shin movement
	
Palate elevation	Finger to nose movement
	
Fundoscopy	Pronator drift
	
Tongue movements	

In a patient with sudden onset of severe occipital or suboccipital pain, the possibility of VADS should be considered however the majority of patients with this symptom will not have VADS. Thus, if neurologic examination is negative, it is reasonable to carefully monitor the patient. If neurologic deficit is noted on exam, VADS should be more strongly suspected and emergency medical attention considered. The imaging modality of choice for suspected VADS is magnetic resonance angiography (MRA) which has good resolution in demonstrating the dissection but, unlike conventional angiography, is non-invasive [9,59]. Doppler ultrasound can also be useful but is generally not as effective as MRA [9].

### Vertebral Artery Dissection and Stroke: The Public Health Message

Public health campaigns have been effectively used for decades to provide important health information to individuals on a wide scale [62-64]. Up to the present, most public discourse regarding the relationship between the chiropractic profession and VADS has revolved around, on the one side, publications [8] and advertising campaigns [65] regarding cervical manipulation being a "risky" treatment with the potential to cause stroke and, on the other side, the chiropractic profession defending the safety of this treatment [66]. However the chiropractic profession now has an opportunity to utilize all that is currently known about VADS to change the discussion from one of defensiveness to one of public health. That is, to engage in a public health campaign to educate the public about the warning signs and symptoms of this uncommon but potentially devastating disorder. While public education materials regarding stroke in general are available from organizations such as the American Stroke Association (http://www.strokeassociation.org/presenter.jhtml?identifier=3030387 accessed 1 April 2010) the National Stroke Association (http://www.stroke.org/site/PageServer?pagename=HOME accessed 1 April 2010) the British Stroke Association (http://www.stroke.org.uk/information/index.html accessed 22 May 2010), the Heart and Stroke Association of Canada (http://www.heartandstroke.com/site/c.ikIQLcMWJtE/b.2796497/k.BF8B/Home.htm?src=home accessed 22 May 2010) and the National Stroke Foundation - Australia (http://www.strokefoundation.com.au/ accessed 22 May 2010) these almost invariably focus on ischemic stroke secondary to arteriosclerosis or hemorrhagic stroke secondary to aneurism or arteriovenous malformation. They do not provide information regarding VADS. Thus, there is no widely available source of information for the public regarding this rare but potentially devastating disorder. Because the chiropractic profession has found itself linked to VADS and because of the paucity of information available to the lay person regarding VADS, it would appear to be beneficial to the profession and, more importantly, the pubic, for Chiropractic Medicine to take the lead on a public education campaign on this topic. A public education campaign specific to VADS would be beneficial on several levels:

1. It would be of benefit to the public as it would provide information regarding a potentially serious disorder that can initially be mistaken for a common, benign condition. Such information is not readily available from other sources, even leading stroke societies.

2. The chiropractic profession has historically taken a defensive approach to the issue of cervical manipulation and stroke. This is certainly understandable given the history of attacks on the profession in this area. However the current understanding of this issue allows the profession to move away from defensiveness toward a positive, proactive, patient-oriented approach. A public health campaign would allow the profession to do this.

3. The chiropractic profession does not have a solid history of involvement in public health [67]. This is evidenced by the relatively small number of members of the Chiropractic Health Section of the American Public Health Association. Because the issue of cervical manipulation and stroke has caused such concern amongst chiropractic physicians over the years, taking a public health approach to this topic may provide the impetus for members of the chiropractic profession to recognize the importance of involvement in public health efforts in general.

There are a number of important points that can be included in a public health campaign regarding VADS:

1. VADS is a rare but potentially serious disorder.

2. Some of the initial symptoms of this disorder can mimic more common and relatively benign neck and headache problems.

3. Because of this, diagnosis can be difficult, so some individuals and their health care providers are not aware that they are experiencing VADS.

4. However there often can be subtle signs and symptoms that may alert a health provider to the possibility of the presence of VADS

5. If you experience any of these signs and symptoms inform your health care provider immediately or call your local emergency service.

The Foundation for Chiropractic Education and Research (which has since disbanded) took the first step in this process by producing a pamphlet for doctors to make available to patients and which provides important information regarding early detection of VADS. A wider campaign can be undertaken by the profession that will bring greater awareness to this disorder.

## Conclusion

The current understanding of the relationship between CMT and VADS provides new responsibilities and new opportunities. The response the chiropractic profession takes to these responsibilities and opportunities will impact its continued maturation and will help to limit suffering among its patients and the public at large. While current evidence suggests that CMT is associated with but not causally related to VADS, it can be expected that patients with undetected VADS will continue to see chiropractic physicians and it is essential that focused attention be made in an attempt at detection of this uncommon but potentially devastating disorder. In addition, the profession would do well to engage in a public health campaign designed to educate the public about VADS to increase recognition of the early signs of this disorder.

## Competing interests

The authors declare that they have no competing interests.
